# The effect of commuting time on burnout: the mediation effect of musculoskeletal pain

**DOI:** 10.1186/s12913-024-10908-1

**Published:** 2024-04-13

**Authors:** Yong-Hsin Chen, Jia June Lin, Ching-wen Yang, Hsiu-Mei Tang, Gwo-Ping Jong, Tsung-Yuan Yang

**Affiliations:** 1 The Department of Health Policy and Management, Chung Shan Medical University, Taichung, 402 Taiwan; 2 Department of Public Health, Chung Shan Medical University, Taichung, 402 Taiwan; 3 Department of Occupational Safety and Health, Chung Shan Medical University Hospital, Taichung, 402 Taiwan; 4 Department of Endocrinology and Metabolism, Chung Shan Medical University Hospital, Taichung, 402 Taiwan; 5 Nursing Department, Chung Shan Medical University Hospital, Taichung, 402 Taiwan; 6 Department of Internal Medicine, Chung Shan Medical University Hospital, Taichung, 402 Taiwan; 7 Institute of Medicine, Chung Shan Medical University, Taichung, 402 Taiwan

**Keywords:** Commuting time, Burnout, Mediation effect, Musculoskeletal pain

## Abstract

**Objectives:**

This study explores the relationship among commuting, musculoskeletal (MS) pain, and burnout.

**Methods:**

An observational and cross-sectional study was conducted at a medical university-affiliated hospital in Taichung, Taiwan in 2021. The two questionnaire was used and they included the Copenhagen Burnout Inventory (CBI) and the Nordic Musculoskeletal Questionnaire (NMQ). All participants were invited to complete the cross-sectional survey. A multiple linear regression was assessed correlations between commuting, MS pain, and burnout.

**Results:**

After excluding those with missing data, 1,615 healthcare workers were deemed valid as research participants. In multiple linear regression, commuting time longer than 50 min was associated with personal burnout (PB) in the presence of adjusted confounders; however, long commuting time was not associated with work-related burnout (WB). Furthermore, the choice of commuting method did not affect PB or WB. Notably, both neck and shoulder pain (NBSP) and ankle pain (BAP) increase the risk of PB and WB. The mediation analysis demonstrated that NBSP is a mediating factor, increasing the level of PB and WB for commuting times longer than 50 min.

**Conclusions:**

Healthcare workers who commute for more than 50 min should be considered part of a high-risk group for burnout and musculoskeletal pain. They should also be provided with resources and programs focused on burnout prevention and MS pain relief.

**Supplementary Information:**

The online version contains supplementary material available at 10.1186/s12913-024-10908-1.

## Introduction

Burnout is described as physical, emotional, and mental exhaustion resulting from long-term involvement in work situations that are emotionally demanding [1]. Notably, developing burnout is a multistage process [2]. In the beginning, people experience at work. Next is the stagnation period, when work pressures cause decreasing enthusiasm. This is followed by the frustration period, when chronic stress gradually causes people to lose enthusiasm and energy for work. Finally, individuals need to seek help and intervention due to poor physical and emotional problems. Symptoms of clinical burnout include emotional exhaustion, physical fatigue, cognitive impairment, disturbed sleep, and functional impairment [3, 4]. As for the factors that impact burnout, previous studies have determined that overtime [5], rotating shift work [6], lack of sleep [6, 7], and chronic diseases [8] are associated with an increased risk of burnout. In contrast, work experience [9], regular exercise habits [7], being married, and being a parent [10] may help decrease burnout levels.

Commuting is a common problem for people living in cities. In Taiwan, the mean commuting time is 23.15 min, with the proportion of commuters spending over 50 min in commuting being 8% [11]. It has been reported that increased commuting time was associated with poor physical and mental health. For instance, individuals who experience long commuting times commonly have poor subjective health and visit the general practitioner[12] more frequently. Moreover, commuting time in compact cities was significantly related to lower satisfaction with life [13]. Conversely, shorter commute times can improve subjective well-being [14].

The proportion of commuters that use public transportation and private vehicles or motorcycles in Taichung city in 2010 were 7.4% and 82.3% [11], respectively. Notably, the method of commuting could be associated with level of satisfaction with life and well-being, and individuals who commute by walking have better mental health [15] and higher life satisfaction [16] than those who drive.

Research has demonstrated that stress caused by commuting was positively related to burnout but had no direct association with job satisfaction [17]. However, the stress of commuting could result from the time spent commuting; for instance, healthcare professionals whose commuting time was more than 30 min seemed to experience higher burnout [18]. However, another study of commuting in Dublin illustrated six modes of transport, including traveling to work by bus, train, car, tram, cycling and walking, which were not associated with burnout [19].

Frequently changing positions from sitting to standing or walking could reduce musculoskeletal pain risk [20]. This result led us to consider whether the long commuting time effect on musculoskeletal (MS) pain resulted from holding the same position for a long time. Some research provided positive evidence to support our opinion, such as, an early study in French nurses found commuting time over one hour was associated with dorsal and lumbar pain [21]. The individuals who report long commuting time easily suffer from MS pain [22]. The risk was 7.29 times higher compared to those who reported short-distance commuting [23]. Coincidentally, not only adults, but also increased commuting times can increase the risk of lower limb dysfunction and low back pain in children [24].

Past studies confirmed that neck or shoulder pain could be associated with mental health. For instance, individuals with low mood/stress [25] and burnout [26] were more likely to develop subsequent neck or shoulder pain. Based on the above, we propose two questions: (1) Does the commuting effect on burnout result from the commuting time, not the commuting method? (2) Is the time spent commuting the main cause of increased burnout resulting from MS pain? To further explore the effect of commuting time on burnout, the present study proposed four hypotheses:

### Hypothesis 1

*The choice of commuting method is not related to an increased risk of burnout.*

### Hypothesis 2

*The effect of commuting time on burnout is significant.*

### Hypothesis 3

*Commuting time is significantly associated with MS pain.*

### Hypothesis 4

*MS pain is a mediating factor between a long commuting time and the increased risk of burnout.*

We believe clarifying the problem could help us understand the cause of the commuting effect on burnout and suggest a full strategy to mitigate burnout in healthcare workers.

## Methods

### Study design

This observational and cross-sectional study was conducted at a medical-university-affiliated hospital in Taichung, Taiwan, between March and April 2021. All 2,531 healthcare workers who have served for one year in the hospital received a QR code by email linking to Google Forms questionnaires. Among them, 1633 (64.52%) individuals filled out full questionnaires. 1,615 (63.81%) of which were deemed valid after excluding those with missing data. The survey included the validated questionnaires of the Copenhagen Burnout Inventory (CBI), the Nordic Musculoskeletal Questionnaire (NMQ), and demographic variables, including family, living habits, work, and physical health. This study was approved by the institutional review board of Chung Shan Medical University in 2021 (No: CS1-21108).

### Measuring burnout

The CBI, developed by researchers from Denmark[27], has very high internal reliability and was formulated to be understandable by and accessible to all people [27]. It has been used to develop three scales, the personal burnout (PB) scale, work-related burnout (WB) scale, and client burnout scale, that can be applied separately to measure burnout in different occupational fields. According to the definition of CBI for PB and WB [27], PB is the degree of physical and psychological fatigue and exhaustion that a person experiences; WB is the degree of physical and psychological fatigue and exhaustion that a person perceives as related to his/her work. To reflect the national conditions, we used the Chinese version of the CBI [28]. Moreover, for the purposes of suitability for all participants, we adopted PB and WB scales to measure burnout for healthcare workers. The scales are listed in Supplementary Information Table S1. The response options of CBI were “always,” “often,” “sometimes,” “seldom,” and ”never/almost never,” which were scored as 100, 75, 50, 25, and 0, respectively. The 13th item in the scales was an inversely scored item (i.e., the responses were scored by minimum “always” = 0 and maximum “never/almost never” = 100, sequentially). The mean of the PB and WB scores (the sum of scores for items 1–6 and items 7–13) represented the level of PB and WB for participants, respectively.

### Measuring musculoskeletal pain

The present study adopted the NMQ, modified and translated by the Taiwan Institute of Occupational Safety and Health. The NMQ measures the presence of pain attributable to work-related factors in the preceding year and is a repeatable, sensitive, and reliable measurement measured of pain [29–31]; when validity tested against clinical history, the result is less than 20% disagreement [32]. The response options of NMQ for the presence of pain sites were the neck, left or right shoulder, upper back, waist or lower back, left or right elbow, left or right wrist, left hip/thigh/buttock, right hip/thigh/buttock, left or right knee, and left or right ankle. The options on frequency of each pain site were every day, once a week, once a month, once every half a year, and at least once every half a year (relative scored as 100, 80, 60, 40, and 20 points).

### The demographic questionnaires

The demographic questionnaires assessed the participants’ age, education degree (response options: “less than high school,” “Bachelor’s degree,” “Master’s degree,” or “PhD.”), marital status (“married” or “other.”), raising children (“without child,” “one child,” “two children,” “three children,” and “over three children.”), weekly exercise habits (at least once per day, at least once weekly, at least once per month, less than once per month, or never), monthly alcohol use habits (every day, occasionally, or never), sleeping time (“<5 h,” “5–6 h,” “6–7 h,” “7–8 h,” or “>8 h.”), overtime work per month (“seldom,” “less than 45 h per month,” “45–80 h per month,” and “more than 80 h per month.”), shift schedules (“day shift work,” “night shift work,” “irregular shift work,” and “regular shift work.”), professional fields (“physicians,” “nurses,” “professional and technical personnel,” and “administrative staff”), and the presence of one or more chronic diseases (“yes” and “no”). Moreover, the participants were asked if they engaged in leisure activities with family or friends during vacation time. The response options, “always,” “often,” “sometimes,” “seldom,” and”never,” were scored as 100, 75, 50, 25, and 0 points, respectively.

Finally, we surveyed participants’ commuting time and most-used commuting methods. The response options for commuting time were “5–10 minutes,” “10–20 minutes,” “20–30 minutes,” “30–40 minutes,” “40–50 minutes,” and “over 50 minutes.” The options of most-used commuting methods (multiple choice) were “train,” “bus,” “Mass Rapid Transit (MRT),” “walking,” bicycle,” “vehicle,” and “motorcycle.”

### Data analysis

The present study adopted four steps to test the four hypotheses presented in the Introduction, as follows.

Step 1: NMQ included complex information for pain sites and occurrence frequency, which is adverse for further statistical analysis. Consequently, we used factor analysis [33] to determine new underlying variables to effectively explain the questionnaire. Factor analysis uses varimax rotation to obtain the standardized scoring coefficients, which constitute new factor loadings. We retained new factors that featured vector values exceeding 1 according to the principle proposed by Hair et al. [33] and redefined new variables according to their corresponding significance. For similar reasons, the questionnaire for the most-used commuter transportation also adopted factor analysis to redefined new variables.

Step 2: We adopted simple linear regression to determine if there was an existing significant difference in the level of burnout among variables.

Step 3: The confounders of PB/WB found in step 2 were adjusting variables and were added to multiple linear regression models of PB/WB. These processes could determine if commuting time, commuting method, and MS pain effect on PB/WB.

Step 4: The present study adopts mediation analysis to determine if the MS pain is a mediating factor for the effect of commuting time on increased burnout. We adopted the strategy proposed by Baron and Kenny, [34] in which (1) the first-stage effect of the independent variable significantly affects the mediating factor; (2) the independent variable significantly affects the dependent variable in the absence of the mediating factor; (3) the second-stage effect of the mediating factor exerts a significant unique effect on the dependent variable; and (4) the effect of the independent variable on the dependent variable weakens upon the addition of a mediating factor to the model. Of them, item (2) is only recommended but not required [35]. The formulas are as follows:

$$ Z=\frac{a \times b}{\sqrt{{b}^{2}{{s}_{a}}^{2}+{a}^{2}{{s}_{b}}^{2}}}$$

where *a* is the linear regression coefficient of the independent variable against the mediating factor, *b* is the linear regression coefficient of the mediating factor against the dependent variable, *c* is the linear regression coefficient of the independent variable against the dependent variable in the absence of mediating factors, and *c’* is the linear regression coefficient of the independent variable against the dependent variable in the presence of a mediating factor. The standard errors of *a* and *b* are represented by s_*a*_ and s_*b*_, respectively. The *Z* exceeding|1.96|,|2.57|, and|3.90| (for a two-tailed test) are significant at α = 0.05, 0.01, and 0.0001, respectively.

Analyses were performed using SAS Enterprise Guide 7.1 software (SAS Institute Inc., Cary, NC, USA), and significance was set at *P* < 0.05.

## Results

Table 1 presents the demographic variables of all 1,615 participants. This is a female-dominated healthcare worker team (women: 81.36%; 1,314 individuals) and 18.39% (297 individuals) of individuals a master’s degree or higher. The average age of all participants was 38.22 ± 10.20. The proportion of participants who are parents (raising at least one child) was 43.49% (703 individuals) of all individuals. The individuals who reported engaging in regular exercise weekly were 57.78% (933 individuals, including at least once per day and at least once weekly). Regarding alcohol use, individuals who report consuming alcohol in a month were 37.71% (609 individuals: including every day and occasionally). Moreover, individuals who report sleeping fewer than 6 h per day were 38.76% (626 individuals: including < 5 h and 5–6 h per day) of all participants. The participants who worked overtime (less than 45 h per month, or 45–80 h per month, or more than 80 h per month) in the past month are 561 individuals, reaching 34.73% of all individuals. Regarding shift work, most shifts were day shift, night shift, irregular shift, and regular shift work, corresponding to 65.70%, 10.28%, 11.89%, and 12.14% of all individuals, respectively. For professional fields, physicians, nurses, professional and technical personnel, and administrative staff were 8.54% (138 individuals), 37.96% (613 individuals), 17.52% (283 individuals), and 35.98% (581 individuals) of all participants. The individuals who suffered from one or more chronic diseases were 39.50% (638 individuals) of all samples. Regarding the frequency of engaging in leisure activities with family or friends, the proportion of responses for always, often, sometimes, seldom, and never were 6.32%, 30.84%, 47.37%, 14.74%, and 0.74% of all individuals, respectively. The highest proportion of commuters who had a commuting time of 10–20 min was 33.50%, followed by 21.42% for 5–10 min. Other commuting times, from high to low, were 10.46% for 30–40 min, 8.67% for > 50 min, and 6.19% for 40–50 min. The most-used transportation methods from high to low were motorcycle (79.07%), private vehicle (32.63%), walking (24.27%), bus or MRT (11.02%), train (8.92%), and bicycle (4.09%).

**Table 1:** The demographic variables of participants

Surveyed variables	Individuals	Proportion (%)/mean ± SD
**Sex**		
Women	1,314	81.36
Men	301	18.64
**Age (years)**	1,615	38.22 ± 10.20
**Education**		
Below high school	81	5.02
Bachelor’s degree	1,237	76.59
Master’s degree	243	15.05
PhD	54	3.34
**Marital status**		
married	779	48.24
other	836	51.76
**Raising children**		
without children	912	56.51
one child	228	14.13
two children	390	24.16
three children	80	4.96
over three children	5	0.25
**Exercise habit**		
at least once per day	154	9.54
at least once weekly	779	48.24
at least once per month	286	17.71
less than once per month	302	18.70
Never	94	5.82
**Alcohol use habit**		
alcohol use every day	8	0.50
alcohol use occasionally	601	37.21
alcohol use never	1,006	62.29
**Sleeping duration**		
< 5 h	63	3.90
5–6 h	563	34.86
6–7 h	719	44.52
7–8 h	232	14.37
> 8 h	38	2.35
**Overtime work per month**		
seldom	1,054	65.26
less than 45 h per month	502	31.08
45–80 h per month	54	3.34
more than 80 h per month	5	0.31
**Shift schedules**		
day shift work	1,061	65.70
night shift work	166	10.28
irregular shift work	192	11.89
regular shift work	196	12.14
**Profession**		
physicians	138	8.54
nurses	613	37.96
professional and technical personnel	283	17.52
administrative staff	581	35.98
**The presence of one or more chronic diseases**		
Yes	638	39.50
No	977	60.50
**Engaging in leisure activities with family and friends during vacation time?**		
always	102	6.32
often	498	30.84
sometimes	765	47.37
seldom	238	14.74
never	12	0.74
**Commuting time (minutes)**		
5–10	346	21.42
10–20	541	33.50
20–30	319	19.75
30–40	169	10.46
40–50	100	6.19
> 50	140	8.67
**The most-used commuter transportation**		
Walking	392	24.27
Bicycle	66	4.09
Bus or MRT	178	11.02
Train	144	8.92
Private vehicle	527	32.63
Motorcycle	1,277	79.07

SD, standard deviation; MRT, Mass Rapid Transit

Table 2 shows that both shoulders (43.09%), neck (36.22%), waist or lower back (27.93%), and upper back (16.90%) were the common MS pain sites of participants. According to the principle proposed by Hair and Anderson (1995) [33], the eigenvalues of Factors 1 and 2, 4.93 and 1.55, respectively, were retained because both exceeded 1. The factor loadings were converted into standardized scoring coefficients through varimax rotation, where the relatively large factor loading values for Factors 1 and 2 corresponded to the MS pain sites of the neck, shoulders, and ankles, respectively. Based on this, we defined Factors 1 and 2 as Neck and Both Shoulders Pain (NBSP) and Both Ankles Pain (BAP).

**Table 2:** MS pain sites and factor analysis of the NMQ

MS pain sites	N	%	Frequency score	Factor loading^1^	
			Mean (SD)	Factor 1	Factor 2
Neck	585	36.22	73.88 (20.71)	0.33	−0.02
Left shoulder	325	20.12	74.89 (22.05)	0.33	−0.01
Right shoulder	371	22.97	76.77 (21.42)	0.33	0.02
Upper back	273	16.90	76.34 (19.96)	0.17	0.00
Waist or lower back	451	27.93	72.33 (23.34)	0.08	−0.04
Left elbow	70	4.33	76.00 (23.98)	−0.05	−0.04
Right elbow	113	7.00	76.11 (24.03)	−0.04	−0.04
Left wrist	77	4.77	77.92 (23.53)	−0.05	0.00
Right wrist	162	10.03	74.82 (23.33)	−0.03	−0.03
Left hip/thigh/buttock	67	4.15	75.22 (21.77)	−0.05	−0.07
Right hip/thigh/buttock	68	4.21	75.29 (22.95)	−0.02	−0.04
Left knee	80	4.95	73.49 (22.11)	−0.05	−0.07
Right knee	88	5.45	76.59 (20.84)	−0.02	−0.04
Left ankle	29	1.80	70.35 (29.09)	−0.02	0.49
Right ankle	25	1.55	71.20 (31.13)	−0.02	0.54
		Eigenvalues		4.93	1.55
		Explained variation %		57.59	18.12

^1^All numbers are standardized scoring coefficients, N, individuals, SD, standard deviation

Table 3 transformed the six most commonly used commuter transportation methods by factor analysis as two new underlying variables to effectively explain the transportation used by individuals: private vehicles or motorcycles (Factor 1) and public transportation system (Factor 2).

**Table 3:** The factor analysis of most-used commuter transportation

The commuter transportation	N	%	Factor loading^1^	
			Factor 1	Factor 2
Walking	392	24.27	−0.005	0.103
Bicycle	66	4.09	0.010	0.006
Bus or MRT	178	11.02	0.005	0.388
Train	144	8.92	0.025	0.323
Private vehicle	527	32.63	−0.441	−0.091
Motorcycle	1,277	79.07	0.475	−0.076
	Eigenvalues		1.074	0.704
	Explained variation %		57.38	37.60

^1^All numbers are standardized scoring coefficients; N, individuals; MRT, Mass Rapid Transit

Table 4 illustrates the statistical association between survey variable and PB/WB by the simple linear regression, where age (B = − 0.25, *P* < 0.0001), weekly exercise habit (B = − 6.30, *P* < 0.0001), monthly alcohol use (B = 3.81, *P* < 0.0001), sleep duration < 6 h per day (B = 8.13, *P* < 0.0001), overtime per month (B = 9.67, *P* < 0.0001), irregular and regular shift work (B = 8.47, *P* < 0.0001; B = 5.20, *P* = 0.0002), physicians (B = 10.60, *P* < 0.0001), nurses (B = 9.07, *P* < 0.0001), PTs (B = 2.50, *P* = 0.049), the presence of chronic diseases (B = 4.69, *P* < 0.0001), engaging in leisure activities with family or friends (LAFF) (B = − 0.12, *P* < 0.0001), NBSP (B = 8.25, *P* < 0.0001), and commuting time of 10–20 min (B = − 2.74, *P* = 0.027) were significantly associated with PB.

**Table 4:** The simple linear regression of survey variable son PB/WB

	PB		WB	
Survey variables	B	P	B	P
**Sex**				
Women	1.84	0.11	1.80	0.08
Men	1.00		1.00	
**Age**	−0.25	< 0.0001	−0.31	< 0.0001
**Education degree**				
Master’s degree or above	0.08	0.946	−2.21	0.034
University or below university degree	1.00		1.00	
**Marriage state**				
Married	−1.06	0.240	−4.30	< 0.0001
Unmarried	1.00		1.00	
**Parenthood**				
Yes	−1.06	0.243	−4.86	< 0.0001
No	1.00		1.00	
**Weekly exercise habit**				
Yes	−6.30	< 0.0001	−5.60	< 0.0001
No	1.00		1.00	
**Monthly alcohol use habit**				
alcohol use ever	3.81	< 0.0001	3.50	< 0.0001
alcohol use never	1.00		1.00	
**Sleeping duration**				
< 6 h	8.13	< 0.0001	6.27	< 0.0001
> 6 h	1.00		1.00	
**Overtime work per month**				
Experience overtime	9.67	< 0.0001	8.88	< 0.0001
Seldom overtime	1.00		1.00	
**Shift schedules**				
Irregular shift work	8.47	< 0.0001	8.87	< 0.0001
Regular shift work	5.20	0.0002	6.23	< 0.0001
Night shift work	2.08	0.161	3.24	0.015
Day shift work	1.00			
**Profession**				
Physicians	10.60	< 0.0001	9.89	< 0.0001
Nurses	9.07	< 0.0001	8.44	< 0.0001
PTs	2.50	0.049	2.17	0.057
ADs	1.00			
**Suffering CD**				
Yes	4.69	< 0.0001	3.47	< 0.0001
No	1.00		1.00	
**Engaging in leisure activities with family or friends**				
LAFF score	−0.12	< 0.0001	−0.14	< 0.0001
**MS pain**				
NBSP	8.25	< 0.0001	6.32	< 0.0001
BAP	1.46	0.006	1.42	0.003
**Commuting time (minutes)**				
> 50	1.89	0.295	−0.96	0.554
40–50	−2.43	0.235	−3.55	0.054
30–40	−2.21	0.192	−3.88	0.011
20–30	−1.46	0.296	−3.44	0.006
10–20	−2.74	0.027	−2.52	0.024
5–10	1.00	-	1.00	-
**The most used commuter transportation**				
Private vehicle or Motorcycle	0.16	0.792	1.06	0.045
Public transportation system	0.06	0.937	0.55	0.397

N, individuals, B, linear regression coefficient, P, p value; PT, professional and technical personnel; AD, administrative staff; CD, chronic diseases

Moreover, the risk or protective factors of WB were age (B = − 0.31, *P* < 0.0001), holding a Master’s degree or above (B = − 2.21, *P* = 0.034), being married (B = − 4.30, *P* < 0.0001), parenthood (B = − 4.86, *P* < 0.0001), weekly exercise habit (B = − 5.60, *P* < 0.0001), monthly alcohol use (B = 3.50, *P* < 0.0001), sleep duration < 6 h (B = 6.27, *P* < 0.0001), experience overtime (B = 8.88, *P* < 0.0001), irregular or regular shift work (B = 8.87; 6.23, both *P* < 0.0001), night shift work (B = 3.24, *P* = 0.015), physicians (B = 9.89, *P* < 0.0001), nurses (B = 8.44, *P* < 0.0001), the presence of chronic diseases (B = 3.47, *P* < 0.0001), engaging in LAFF (B = − 0.14, *P* < 0.0001), NBSP (B = 6.32, *P* < 0.0001), BAP (B = 1.42, *P* = 0.003), commuting time of 30–40 min (B = − 3.88, *P* = 0.011), commuting time of 20–30 min (B = − 3.44, *P* = 0.006), commuting time of 10–20 min (B = − 2.52, *P* = 0.024), and private vehicle or motorcycle (B = 1.06, *P* = 0.045).

Table 5 mainly show if different commuting times or commuter transportation methods impact PB and WB in the presence of adjusted confounders. According to M_1_, M_2_, and M_3_ models in Table 5, commuting times longer than 50 min were significantly associated with increased PB (B = 4.21, *P* = 0.015; B = 3.39, *P* = 0.035; B = 4.24, *P* = 0.015). Moreover, we also found the NBSP effect could explain 19.48% ($=\frac{3.39-4.21}{4.21}$) of the residual effects of commuting time < 50 on PB according to the M_2_ model and itself keep significant in statistic (B = 7.09, *P* < 0.0001). Nevertheless, the BAP effect on PB is only slightly affected by commuting times longer than 50 min (B value for commuting time < 50 changed from 4.21 to 4.24) according to the M_3_ model. Overall, NBSP may play an important role between commuting times longer than 50 min and increased PB. In addition, regarding commuter transportation effect on PB, M_1_, M_2_, and M_3_ models in Table 5, it was determined that commuter transportation was not associated with increased levels of PB (*P* < 0.05) in the presence of adjusted variables.

**Table 5:** The multiple linear regression of commuting time or commuter transportation effect on personal and work-related burnout

	PB						WB					
	M_1_		M_2_		M_3_		M_1_		M_2_		M_3_	
Survey variables	B	P	B	P	B	P	B	P	B	P	B	P
**Commuting time**												
> 50	4.21	0.015	3.39	0.035	4.24	0.015	1.82	0.242	1.22	0.406	1.85	0.233
40–50	−0.37	0.848	−1.12	0.947	−0.55	0.776	−0.97	0.575	−0.80	0.627	−1.15	1.73
30–40	1.38	0.384	1.47	0.316	1.30	0.410	0.03	0.981	0.12	0.930	−0.04	0.978
20–30	1.78	0.175	2.02	0.097	1.78	0.176	0.11	0.928	0.37	0.744	0.09	0.938
10–20	−0.41	0.720	−0.34	0.745	−0.48	0.672	−0.33	0.749	−0.30	0.755	−0.40	0.699
5–10	1.00	-	1.00	-	1.00	-	1.00	-	1.00	-	1.00	-
**The most used commuter transportation**												
Private vehicle or Motorcycle	−0.03	0.965	0.96	0.298	−0.09	0.885	0.32	0.541	0.70	0.162	0.28	0.603
Public transportation system	0.05	0.943	0.17	0.786	−0.09	0.890	0.64	0.289	0.66	0.252	0.50	0.405
**MS pain**												
NBSP	-	-	7.09	< 0.0001	-	-	-	-	5.45	< 0.0001	-	-
BAP	-	-	-	-	1.23	0.011	-	-	-	-	1.24	0.004

B, linear regression coefficient; P, p value; For PB, M_1_, the model adjusted commuting time, the most used commuter transportation, age, regular exercise every week, alcohol use habit in a month, sleep duration, overtime work per month, shift schedules, profession, suffering CD, and engaging in leisure activities with family or friends; M_2_, M_1_ extra adjusted NBSP; M_3_, M_1_ extra-adjusted BAP. For WB, M_1_, the model adjusted commuting time, the most used commuter transportation, age, education degree, marriage state, parenthood, regular exercise every week, alcohol use habit in a month, sleep duration, overtime work per month, shift schedules, profession, suffering CD, and engaging in leisure activities with family or friends; M_2_, M_1_ extra adjusted NBSP; M_3_, M_1_ extra-adjusted BAP

Table 5 shows that regardless of the model (M_1_, M_2_, or M_3_), commuting time and the commuter transportation method used were not associated with increased levels of WB (*P* > 0.05). In contrast with the effect on PB, commuting time is not associated with increased WB.

Based on the above results, we confirmed Hypothesis 1 that the choice of commuter transportation is unrelated to increased burnout. The present study also confirmed Hypothesis 2 that the effect of commuting time on burnout is significant in the presence of adjusting confounders. Nevertheless, we emphasize that only commuting times longer than 50 min are associated with increased burnout; furthermore, the feeling of burnout comes from personal reasons, not work-related reasons.

We found that commuting times longer than 50 min were associated with increased risk of NBSP (Table 6: B = 0.21, *P* < 0.01) but not BAP (B = − 0.02, *P* = 0.802; B = 0.02, *P* = 0.414). The statistical results confirm Hypothesis 3 that commuting time is significantly associated with MS pain. Additionally, Table 7 also demonstrated the commuting in private vehicles or motorcycles were associated with reduced risk of NBSP (Table 8: B = − 0.10, *P* = 0.001). A more valuable finding was that commuting times longer than 50 min increased the risk of MS pain limited to the neck and shoulders.

**Table 6:** The association between MS pain and commuting time or commuting methods used

Survey variables	NBSP		BAP	
	B	P	B	P
Commuting time > 50 vs. others	0.21	0.010	−0.02	0.802

B, linear regression coefficient, P, p value

**Table 7:** The mediation effect of NBSP for commuting times longer than 50 min increasing WB

	Dependent variable: WB		Mediating factor: NBSP				
Independent variable	c	c’	a	s_a_	b	s_b_	Z
Commuting time > 50 vs. others	1.39	0.07	0.21^**^	0.081	6.32^***^	0.413	2.56
Private vehicle or Motorcycle	1.03^*^	1.67^**^	−0.10^**^	0.030	6.43^***^	0.412	−3.26

^*^, P < 0.05; ^**^, P < 0.001; ^***^, P <.0001; c, the linear regression coefficient of independent variable against dependent variable in absence of mediating factor; c,’ the linear regression coefficient of independent variable against dependent variable in presence of mediating factor; a, the linear regression coefficient of independent variable against mediating factor; b, the linear regression coefficient of mediating factor against dependent variable; s_a_; the standard errors of a; s_a_; the standard errors of b

**Table 8:** The mediation effect of NBSP for commuting time over 50 min increasing PB

	Dependent variable: PB		Mediating factor: NBSP				
Independent variable	c	c’	a	s_a_	b	s_b_	Z
Commuting time > 50 vs. others	3.63^*^	1.90	0.21^**^	0.081	8.22^***^	0.445	2.57

^*^, P < 0.05; ^**^, P < 0.001; ^***^, P <.0001; c, the linear regression coefficient of independent variable against dependent variable in absence of mediating factor; c,’ the linear regression coefficient of independent variable against dependent variable in presence of mediating factor; a, the linear regression coefficient of independent variable against mediating factor; b, the linear regression coefficient of mediating factor against dependent variable; s_a_; the standard errors of a; s_a_; the standard errors of b

Based on strategies proposed by Baron and Kenny [34], only NBSP confirms the first-stage effect (Table 6, B = 0.21, *P* = 0.010) and the second-stage effect (Table 8, b = 8.22, *P* < 0.001), but BAP does not. Therefore, NBSP will be included in a shortlist of mediating factors. Tables 7 and 8 determined NBSP was a mediating factor (Z = 2.57, *P* < 0.01; Z = 2.56, *P* < 0.01) of commuting times longer than 50 min increasing the risk of PB and WB. Notably, NBSP also was a suppression factor between private vehicles or motorcycles and WB (Table 7, Z = − 3.26, *P* < 0.01). Namely, commuting in private vehicle or motorcycle reduced neck and both shoulders pain relative to other commuter methods that mitigated WB increases.

BAP did not satisfy the first-stage effect (Table 6, B = − 0.02, *P* = 0.802) strategies proposed by Baron and Kenny [34], thus, it is excluded from the shortlist of mediating factors.

According to the above results, we confirmed Hypothesis 4 that MS pain is a mediating factor between long commuting times and increased risk of burnout. However, for the mediation effect, the sites of the MS pain effect on burnout are specifically associated with neck and both shoulders pain. Another important finding is that commuting times over 50 min indicate that long commuting times led to MS pain, further affecting the risk of increased PB and WB.

## Discussion

Past research had identified many factors that contribute to burnout. For instance, individuals who experience overtime [5], rotating shift work [6], lack of sleep [6, 7], and suffer from chronic diseases [8] suffer a higher risk of burnout than others. Similarly, the present study found that individuals who experience overtime had irregular or regular shift work, slept fewer than 6 h, and had chronic diseases had significantly higher levels of PB and WB than others. Notably, some research also identified that factors associated with reduced burnout include work experience [9], regular exercise habits [7], being married, having children [10], etc. These similar protective factors also were found in our study, for instance, individuals who had a weekly exercise habit had lower levels of PB and WB than those who reported no weekly exercise habit. Moreover, our study also confirmed that married individuals or parents reported lower WB levels than others.

Moreover, evidence demonstrated burnout was strongly associated with alcohol use among healthcare workers such as physicians, nurses, and residents [36, 37]. Our study found a relationship between burnout and alcohol use: individuals who have ever used alcohol in a month reported higher levels of PB and WB than others. Notably, practicing physicians have a greater prevalence of burnout than individuals in other fields [38, 39]. We observed a similar result among participants: physicians experience a markedly personal and work-related burnout compared with other profession fields.

For stressed individuals, engaging in leisure activities can relieve stress, improve emotional health, and maintain physical and mental health [40–42]. Our study also confirmed this result. Table 4 shows that positively engaging in leisure activities with family or friends was significantly associated with reduced PB and WB.

Musculoskeletal (MS) pain could be associated with burnout. Some studies have suggested that neck or shoulder pain is associated with low mood/stress [25] and burnout more likely leads to neck or shoulder pain [26]. Coincidentally, our study also presents the same findings; after adjusting for confounders, neck/shoulder pain and ankle pain were found to be associated with increased risk of personal burnout (PB) and work-related burnout (WB). Moreover, burnout increases the risk of MS pain because of the activation of the autonomic nervous system and the hypothalamic–pituitary–adrenal axis [43].

Regarding the relationship between the choice of commuter transportation and burnout, a study in Hong Kong on commuting and well-being in 2015 demonstrated that the commuting method was not an independent risk factor for well-being [13]. Although private vehicle or motorcycle use was associated with a high risk of WB (Table 4), the association was not maintained after adjusting for confounders (Table 5).

In a Taiwanese study, 8% of individuals had commuting times > 50 min [11], compared with our study in which 8.67% had commuting times > 50 min, which is slightly higher than the country’s average. Commuting time is associated with subjective health [12], well-being [14], and satisfaction with life [13]. Regarding the influence of family life, studies have demonstrated that individuals have less energy after commuting, which can affect their quality of life [44] because longer commuting times tend to disrupt work and family life [45]. These factors lead to a higher sense of family responsibilities [46], reduced time for leisure activities [47], and work–family conflict [48]. Based on these results, does the effect of commuting time on individuals and their families directly or indirectly affect burnout? Using multiple linear regression M1, M2, and M3 models, individuals who reported having commuting times > 50 min sustained a higher risk of PB than others. However, commuting times did not affect WB development despite adjustments for confounders. Long commuting times (such as > 50 min) directly affected PB but not WB.

Although commuting time influenced burnout development (hypothesis 2), whether it was a beneficial or detrimental factor was dependent on whether commuting was a source of stress or relaxation. Specifically, commuting is not always a source of stress because it might be a form of mental relaxation and a protective screen between work and family [49]. A similar effect was observed in this study. For instance, Table 4 demonstrates that all commuting times < 40 min are associated with reduced WB risk, demonstrating that individuals who reported commuting times < 40 min sustained less risk of WB than those who reported commuting times between 5 and 10 min. In addition, individuals who reported commuting times between 10 and 20 min sustained a low risk of burnout. Thus, if commuting relaxes an individual mentally or emotionally, commuting times can be important. Overall, we found that commuting times > 50 min will not mitigate burnout but will worsen it. Therefore, whether commuting is a source of stress or a form of mental relaxation will depend on the commuting time.

This study also confirmed the relationship between commuting time and MS pain (hypothesis 3); moreover, evidence showed that a longer commuting time was associated with an increased risk of MS pain. For instance, a study of railway workers in 2015 demonstrated that individuals who experienced commuting time > 60 min had a higher number of complaints of MS pain than those who experienced commuting time < 60 min [22]. A study of full-time bank employees in Dhaka City between December 2018 and May 2019 demonstrated that the proportions of individuals who experienced MS pain and reported commuting times > 60 min or 31–60 min were 7.29 and 6.35 times higher, respectively, than those who reported commuting time < 15 min [23]. Compared with previous studies, 50 min was the cut-off point for MS pain, which links this relationship with burnout, MS pain, and commuting time. Table 6 shows that commuting times > 50 min were associated with increased NBSP, which is consistent with the results of previous studies. A study in adults illustrated that increased commuting distance was associated with a higher risk of physical inactivity [50]. Moreover, sedentary workers have an increased risk of MS pain [51]. Notably, a study of commuting methods for children found that decreased walking duration and increased sitting duration associated with vehicle commuting induced low back pain [24]. Therefore, sedentary behavior may be associated with long commuting times and increased risk of MS pain [20].

We tested hypotheses 1–3 and found that MS pain was associated with commuting time and burnout. Nevertheless, the above relationships lead to a new question about whether MS pain plays a key role in the relationship between long commuting times and increased risk of burnout. We adopted mediation analysis to answer this question. Tables 7 and 8 demonstrate that NBSP is a mediating factor and that commuting time > 50 min increases the risk of PB and WB. Accordingly, healthcare workers who commute for > 50 min are more likely to experience neck and shoulder pain, which may further intensify burnout. Therefore, sedentary behaviors should be avoided during commutes. In addition, hospitals should include individuals with long commutes in the high-risk group for burnout and provide resources and training programs to prevent or mitigate MS pain. To the best of our knowledge, this is the first study to report these findings.

Our study has some limitations. MS pain can be the result of workload, work styles, or posture. Unfortunately, our study did not collect such data in the regression models. Notably, we were unable to determine whether high work stress or emotional exhaustion due to the pandemic affected the findings; thus, a similar study during the nonpandemic period should be replicated and the results compared with the pandemic period. In addition, past studies have shown that working long hours or working overtime does pose a high risk of burnout [5]. Table 4 found that overtime was a risk factor for PB. In addition, commuting time of more than 50 min remained a risk factor for PB in the presence of adjusted variables including overtime (Table 5, M_2_, B = 3.39, *P* = 0.035; M_3_ = 4.24, *P* = 0.015). Therefore, excluding the effect of long working hours on burnout, long commute times still affect burnout. Whether overtime or long working hours are mediating factors needs to be determined in future studies.

Because the mediation model of an observational study could be biased[52], as causal relationships show a higher risk of judgment. Therefore, we do not conclude a “causal relationship” in our conclusion to avoid misleading readers.

## Conclusion

The present study suggests the commuting method chosen is not associated with increased PB and WB. Specifically, commuting times over 50 min will obviously increase the risk of personal burnout, but work-related burnout is not affected. In addition, healthcare workers who commute for > 50 min are at a higher risk than others for neck and shoulder pain, which were associated with increased PB and WB levels. Therefore, healthcare workers with long commuting times should be considered a high-risk group for burnout and MS pain. They should also be provided with resources and programs focused on burnout prevention and MS pain relief.

### Electronic supplementary material

Below is the link to the electronic supplementary material.

Supplementary Material 1

## Data Availability

Datasets used and/or analyzed during the current study are available from the corresponding author on reasonable request.
