# Supplementary material for: The effect of commuting time on burnout: the mediation effect of musculoskeletal pain
Source: BMC Health Serv Res. 2024 Apr 13;24:468. doi: 10.1186/s12913-024-10908-1 (PMC11016201; doi:10.1186/s12913-024-10908-1)
Supplement: Supplementary file 1 — Supplementary Material 1 [file 12913_2024_10908_MOESM1_ESM.docx]

**Supplementary Information**

Table S1. The 13 items for the PB and WB scales

| The first six items, which concern PB, are as follows: |
| --- |
| 1 “How often do you feel tired?”  2 “How often are you physically exhausted?”  3 “How often are you emotionally exhausted?”  4 “How often do you think “I can’t take it anymore?”  5 “How often do you feel worn out?”  6 “How often do you feel weak and susceptible to illness?” |
| Items 7–13, which concern WB, are as follows: |
| 7 “Is your work emotionally exhausting?”  8 “Do you feel burned out because of your work?”  9 “Does your work frustrate you?”  10 “Do you feel worn out at the end of the working day?”  11 “Are you exhausted in the morning at the thought of another day at work?” |
